# CDHu40: a novel marker gene set of neuroendocrine prostate cancer

**DOI:** 10.1093/bib/bbae471

**Published:** 2024-09-25

**Authors:** Sheng Liu, Hye Seung Nam, Ziyu Zeng, Xuehong Deng, Elnaz Pashaei, Yong Zang, Lei Yang, Chenglong Li, Jiaoti Huang, Michael K Wendt, Xin Lu, Rong Huang, Jun Wan

**Affiliations:** Department of Medical and Molecular Genetics, Indiana University School of Medicine, 410 W 10th Street, Indianapolis, IN 46202, United States; Borch Department of Medicinal Chemistry and Molecular Pharmacology, Purdue University, 575 Stadium Mall Drive, West Lafayette, IN 47907, United States; Department of Biological Sciences, Boler-Parseghian Center for Rare and Neglected Diseases, Harper Cancer Research Institute, University of Notre Dame, 100 Galvin Life Science Center, Notre Dame, IN 46556, United States; Borch Department of Medicinal Chemistry and Molecular Pharmacology, Purdue University, 575 Stadium Mall Drive, West Lafayette, IN 47907, United States; Department of Medical and Molecular Genetics, Indiana University School of Medicine, 410 W 10th Street, Indianapolis, IN 46202, United States; Department of Biostatistics & Health Data Science, Indiana University School of Medicine, 410 W 10th Street, Indianapolis, IN 46202, United States; Department of Pediatrics, Herman B Wells Center for Pediatric Research, Indiana University School of Medicine, 1044 W Walnut St, Indianapolis, IN 46202, United States; Department of Medicinal Chemistry, College of Pharmacy, University of Florida, 1345 Center Dr Room P3-12, Gainesville, FL 32603, United States; Department of Pathology, Duke University School of Medicine, Davison Building, 40 Duke Medicine, Durham, NC 27710, United States; Department of Internal Medicine, Division of Hematology and Oncology, University of Iowa, 200 Hawkins Dr, Iowa City, IA 52242, United States; Holden Comprehensive Cancer Center, University of Iowa, 200 Hawkins Dr, Iowa City, IA, 52242, United States; Department of Biological Sciences, Boler-Parseghian Center for Rare and Neglected Diseases, Harper Cancer Research Institute, University of Notre Dame, 100 Galvin Life Science Center, Notre Dame, IN 46556, United States; Indiana University Simon Comprehensive Cancer Center, Indiana University School of Medicine, 535 Barnhill Dr, Indianapolis, IN 46202, United States; Borch Department of Medicinal Chemistry and Molecular Pharmacology, Purdue University, 575 Stadium Mall Drive, West Lafayette, IN 47907, United States; Department of Medical and Molecular Genetics, Indiana University School of Medicine, 410 W 10th Street, Indianapolis, IN 46202, United States; Indiana University Simon Comprehensive Cancer Center, Indiana University School of Medicine, 535 Barnhill Dr, Indianapolis, IN 46202, United States; Center for Computational Biology and Bioinformatics, Indiana University School of Medicine, 410 W 10th Street, Indianapolis, IN 46202, United States

**Keywords:** NEPC, biomarker, protein–protein interaction (PPI)

## Abstract

Prostate cancer (PCa) is the most prevalent cancer affecting American men. Castration-resistant prostate cancer (CRPC) can emerge during hormone therapy for PCa, manifesting with elevated serum prostate-specific antigen levels, continued disease progression, and/or metastasis to the new sites, resulting in a poor prognosis. A subset of CRPC patients shows a neuroendocrine (NE) phenotype, signifying reduced or no reliance on androgen receptor signaling and a particularly unfavorable prognosis. In this study, we incorporated computational approaches based on both gene expression profiles and protein–protein interaction networks. We identified 500 potential marker genes, which are significantly enriched in cell cycle and neuronal processes. The top 40 candidates, collectively named CDHu40, demonstrated superior performance in distinguishing NE PCa (NEPC) and non-NEPC samples based on gene expression profiles. CDHu40 outperformed most of the other published marker sets, excelling particularly at the prognostic level. Notably, some marker genes in CDHu40, absent in the other marker sets, have been reported to be associated with NEPC in the literature, such as DDC, FOLH1, BEX1, MAST1, and CACNA1A. Importantly, elevated CDHu40 scores derived from our predictive model showed a robust correlation with unfavorable survival outcomes in patients, indicating the potential of the CDHu40 score as a promising indicator for predicting the survival prognosis of those patients with the NE phenotype. Motif enrichment analysis on the top candidates suggests that REST and E2F6 may serve as key regulators in the NEPC progression.

## Introduction

Prostate cancer (PCa) is the most common cancer among American men, with an estimated 288 300 new diagnoses projected for 2023 [1]. Treatments of PCa include surgery, radiotherapy, chemotherapy, hormone therapy, and immunotherapy. However, the development of castration-resistant prostate cancer (CRPC) during hormone therapy poses a significant challenge. CRPC is characterized by sustained high serum prostate-specific antigen (PSA) levels, ongoing disease progression, and potential metastasis to the new sites, leading to a poor prognosis [2–4]. Most CRPC tumors still depend on androgen receptor (AR) signaling, and therefore, the use of current androgen-signaling inhibitors (ASIs) such as enzalutamide (ENZ) and abiraterone can offer temporary relief from resistance [5]. However, a subset of the CRPC patients exhibited neuroendocrine (NE) phenotype with diminished or absent reliance on AR signaling and a dismal prognosis [6–8]. The most lethal subtype of this disease, however, has similar initial symptoms comparable to CRPC and hence lacks appropriate unique identification markers. NE PCa (NEPC) biopsy samples also often exhibit in mixed histology, posing formidable challenges on accurate diagnosis and appropriate treatments [9].

Thus, developing accurate diagnosis and imaging tools for NEPC is the crucial first step in effectively managing the disease. Numerous studies have attempted to identify the most frequently overlapping markers for NEPC patients including delta-like ligand 3 (DLL3), with high expressions exclusively in CRPC-NE cells [10]. A clinical trial is in progress at Memorial Sloan Kettering Cancer Center to evaluate DLL3 PET imaging in patients with small cell lung cancer (SCLC) and NEPC (NCT04199741). Additional immunohistochemistry (IHC) of canonical NEPC marker genes, such as CHGA, SYP, NCAM1, ENO2, AR, and KLK3, can be used to clinically identify NEPC samples [11], however, the IHC of these markers may not always be directly applicable [12]. Given the feasibility of gene expression profiles with the development of next-generation sequencing (NGS) technologies, an alternative option is to identify NEPC based on the expression levels and/or other information of gene markers, exploring marker genes beyond the six canonical NEPC markers (Supplementary Table S1 available online at http://bib.oxfordjournals.org/).

Despite these efforts, protein–protein interactions (PPIs) have not yet been considered in the selection of NEPC biomarkers in these reports. PPIs are known to form a fundamental network and are integral to almost all biological processes, the alteration of which contributes to disease progression. In our study, we incorporated aberrant gene expression and the PPI information by applying the method of using knowledge in network (uKIN) [13] for PPI analysis to explore biomarkers effectively distinguishing NEPC from all of the PCa samples. Our approach facilitates the identification of robust biomarkers associated with NEPC, unveiling novel markers not previously reported as NEPC biomarkers by traditional methods. The top 40 marker genes, denoted as CDHu40, exhibited a remarkable accuracy in predicting NEPC and demonstrated a strong correlation with patient survival. Taken together, our results highlight the potential significance of CDHu40 as a prognostic indicator for NEPC.

## Methods

### Gene expression data sets used in this study

Eight data sets with defined NEPC samples were selected in this study (Table 1). NEPC_WCM_2016 and PRAD_SU2C_2019 were used for the training and validation sets. Three data sets, GSE32967, GSE149091, and GSE59984, were downloaded from the Gene Expression Omnibus (GEO) database being used as independent test sets on our model. To our knowledge, samples in prostate adenocarcinoma (PRAD) from the The Cancer Genome Atlas Program (TCGA) (PRAD_TCGA) were all primary tumors that had barely NEPC cases. So, PRAD_TCGA was used as a negative control to estimate the false positives of the NEPC samples by different marker gene sets.

**Table 1:** Data sets used in this study.

Data set	Data type	Number of samples			Reference (PMID or link to the datasets)
		Total	NEPC	Non-NEPC^a^	
WCM_NEPC_2016 [6]	Bulk RNA-seq	49	15	34	PMID: 26855148
PRAD_SU2C_2019 [14]	Bulk RNA-seq	232	22	210	PMID: 31061129
GSE32967 [15]	Microarray	22	14	8	PMID: 22156612
GSE149091 [16,17]	Bulk RNA-seq	4	1	3	PMID: 32531951, PMID: 32512818
GSE59984 [18]	Microarray	14	2	12	PMID: 29757368
PRAD_TCGA	Bulk RNA-seq	498	0	498	https://www.cancer.gov/tcga
Asberry *et al.* 2022 [19]	scRNA-seq	4	3	1	PMID: 36382181
Dong *et al.* 2020 [20]	scRNA-seq	5	4	1	PMID: 33328604

a Non-NEPC represents PCa samples collected in studies that were not recognized as NEPC.

The expression value of samples in GSE32967 was retrieved using getGEO function. Gene expressions in GSE149091 and GSE59984 were downloaded from the Supplementary file section in the GEO entry. The expression values were standardized before feeding to the prediction model. Transcript per millions (TPM) expression value of samples in Lundberg *et al*. [21] were downloaded from the repository provided in the paper with log_2_ transformation for further analysis. Two more scRNA-seq data sets, Asberry *et al*. [19] and Dong *et al*. [20], were used to test our model at the single cell level. Specifically, raw counts were downloaded from GSE215943 and GSE137829, respectively. Cells with unique gene counts over 8000 or having >10% mitochondrial genes were filtered out. The gene expression levels were normalized by total expression of the cell, multiplied by a scaling factor 10 000, and then transformed using log_2_. Samples were integrated using FindIntegrationAnchors [22]. After scaling the integrated data, the first 30 principal components from principal component analysis were used to cluster the cells by a shared nearest neighbor (SNN) modularity optimization–based clustering algorithm. The algorithm first calculates *k*-nearest neighbors and constructs the SNN graph. Then, the modularity function was optimized to determine clusters using the Louvain algorithm. UMAP (Uniform Manifold Approximation and Projection for Dimension Reduction) visualizations were performed with Seurat package. The NE phenotype of cell clusters was derived according to the expression levels of canonical marker genes. For scRNA-seq data analysis, if genes of interest had missing expression data in cells, we assume that the data are missing at random and assign them an expression value of 0 in model construction and prediction.

### Identification of differentially expressed genes

Messenger RNA (mRNA) gene expression profiles from the first two datasets in Table 1, NEPC_WCM_2016 [6] and PRAD_SU2C_2019 [14], were retrieved from cBioportal [23,24], followed by log_2_ transformation. Limma [25] was used to identify differentially expressed genes (DEGs) with the cutoffs of False Discovery Rate (FDR) < 0.05 and |log_2_FC| > 1 between NEPC and non-NEPC samples for two datasets, respectively, given the sample information from the above datasets. The average amplitude of log_2_FC from the comparisons in the two datasets were used as the input of the analysis of association of NEPC genes.

### Inference of neuroendocrine prostate cancer–associated genes using knowledge in network

Genes associated with a particular disease usually target a limited number of pathways and are often clustered together in the network. Therefore, propagating disease relatedness within the network is an effective approach for identifying disease-related genes. Unlike other network analysis methods, uKIN [13] leverages prior knowledge of disease-associated genes to guide random walks on a known physical PPI network starting from potential disease-associated genes identified through differential gene expression analysis. Specifically, weights for all genes in the PPI network were derived by injecting fluid originating from known disease-associated genes, influencing the random walk. Higher weights indicate greater likelihood of the gene transitioning to neighboring genes within the network. Ultimately, the accumulated fluid at each gene after the random walk processing is represented by its score. In our study, the uKIN [13] was utilized to discover NEPC-related genes strongly associated with the six canonical NEPC marker genes commonly used to clinically identify the NEPC sample: AR, PSA (KLK3), CHGA, SYP, CD56 (NCAM1), and NSE (ENO2) [11], which were adopted as seeds in the uKIN analysis. The PPI information was retrieved by physical interaction in the StringDB [26]. DEGs between NEPC and non-NEPC samples were considered as new information related to NEPC to guide the random walk. The amplitudes of gene expression fold changes (FCs) in log_2_ scale (|log_2_FC|) between NEPC and non-NEPC were added as the weights of genes for the uKIN analysis. The parameter α for the uKIN was set to 0.5 as restart probability, whereas the flow rate, γ, was set to 1.

### Logistic regression model for neuroendocrine prostate cancer classification

Glmnet [27] was used to generate a logistic regression model to classify NEPC and non-NEPC samples based on marker genes selected. Optimal parameters of the model are estimated using cross-validation incorporating elastic net penalty. Beltran *et al*. [6] and Abida *et al*. [14] data were randomly split into training sets including 28 NEPC and 183 non-NEPC samples and test sets (9 NEPC and 61 non-NEPC samples). A logistic regression model was built using cross-validation on the training set. The resulting model was tested on the test set and the other three independent GEO test sets (Table 2). The performance of prediction was evaluated based on the area under the precision–recall curve (AUPRC).

**Table 2:** Primers used for qPCR.

Gene	Forward primer	Reverse primer
GAPDH	GAAGGTGAAGGTCGGAGTC	GAAGATGGTGATGGGATTTC
AR	GACGACCAGATGGCTGTCATT	GGGCGAAGTAGAGCATCCT
ASCL1	CCCAAGCAAGTCAAGCGACA	AAGCCGCTGAAGTTGAGCC
ENO2	AGCCTCTACGGGCATCTATGA	TTCTCAGTCCCATCCAACTCC
SYP	CTCGGCTTTGTGAAGGTGCT	CTGAGGTCACTCTCGGTCTTG
CHGA	TAAAGGGGATACCGAGGTGATG	TCGGAGTGTCTCAAAACATTCC
DDC	TGGGGACCACAACATGCTG	TCAGGGCAGATGAATGCACTG
BEX1	GCAGTAAACAGTCTCAGCATGG	GGCTCCCCTTTATTAGCAACTT
HGFAC	GTGTGCCACAACTCACAACTA	GGTCCTGGGTATTGGAGCA
MAST1	TCTCTGGACCGCGCTTTCTA	TGAGGCTTTTCCGATTACTGGT
CACNA1A	CGCTTCGGAGACGAGATGC	TGCGCCATTGACTGCTTGT
FOLH1	CCATTAGGGTTACCAGACAGGC	CCCTGCATACTTGTTGTGGC
CPNE4	ATGAGCAACATTTATGAGTCCGC	CTGCCCATGAGACTGCATCT
RBP4	AGGAGAACTTCGACAAGGCTC	GAGAACTCCGCGACGATGTT
ALB	TGCAACTCTTCGTGAAACCTATG	ACATCAACCTCTGGTCTCACC
FGB	AGTGATTCAGAACCGTCAAGAC	CATCCTGGTAAGCTGGCTAATTT
FGG	TTATTGTCCAACTACCTGTGGC	GACTTCAAAGTAGCAGCGTCTAT
NCAM1	GGCATTTACAAGTGTGTGGTTAC	TTGGCGCATTCTTGAACATGA

### Survival analysis

Survival probability was computed using the R function survfit. The pathological grade is an important potential confounding factor affecting survival outcomes, so we adjusted it as a stratified factor in the baseline hazard using the stratified survival analysis. Since no stage information was available for the PRAD_SU2C_2019 dataset, we categorized the grade based on the Gleason score: low grade for the Gleason score of 6, intermediate grade for the Gleason score of 7, and high grade for the Gleason score of 8–10. The significance of the difference in survival times of different groups was determined by log-rank test by Cox regression, and the low score group was used as the reference group to calculate the hazard ratio. The Kaplan Meier (KM) plots were generated using the ggsurvplot function in the package survminer [28,29]. The summary of the metadata of the samples used in survival analysis is shown in Supplementary Tables S2 and S3 available online at http://bib.oxfordjournals.org/.

### Functional analysis of top candidate genes

The top 500 NEPC candidate marker genes from our results were entered into StringDB [26] for visualization. The functional enrichment analysis was performed using The Database for Annotation, Visualization and Integrated Discovery (DAVID) [30,31] on top 500 genes with increased and decreased expression in NEPC samples, respectively.

### Cell culture

androgen-sensitive human prostate adenocarcinoma cells derived from the left supraclavicular lymph node metastasis (LNCaP: CRL-1740) and NCI-H660 (CRL-5813) were purchased from American Type Culture Collection (atcc.org). The KUCaP13 cell line was obtained from the laboratory of Shusuke Akamatsu at Kyoto University [32]. LNCaP and KUCaP13 cells were cultured in RPMI-1640 medium supplemented with 10% fetal bovine serum (FBS), 1% penicillin–streptomycin (Pen-Strep), and 1% HEPES. NCI-H660 cells were cultured in RPMI-HITES medium containing 5% FBS, 0.005 mg/ml insulin, 0.01 mg/ml transferrin, 30 nM sodium selenite, 10 nM hydrocortisone, 10 nM beta-estradiol, extra 2 mM L-glutamine, and 1% Pen-Strep. LNCaP was passaged at a 1:5 ratio every 3–5 days. For NCI-H660 and KUCaP13, half of the medium was refreshed twice a week and passaged when cell concentration exceeded 1 × 10^6^ cells/ml. All cell cultures were incubated at 37°C with 5% CO_2_ and assessed for mycoplasma monthly by The polymerase chain reaction (PCR). All mycoplasma results were negative.

### Gene expression detection by quantitative PCR (qPCR)

We further validated the expression levels of 17 selected NEPC markers and GAPDH by qPCR (Table 2) in LNCaP, NCI-H660, and KUCaP13 cells. Total RNA was extracted from cells with EZ-10 Spin Column Animal Total RNA Miniprep Kit (Bio Basic, BS82312), and complementary DNA (cDNA) was synthesized using All-In-One 5X RT MasterMix (Applied Biological Materials, G592) with 500 ng/μl RNA template, following the respective manufacturer protocols. cDNA was amplified with 2× SYBR Green qPCR Master Mix (Bimake, B21202), and qPCR reaction was run on Bio-Rad CFX Connect system with the following conditions: 95°C for 10 min, followed by 40 cycles with 15 s at 95°C, and 60 s at 60°C, and a final dissociation curve step with 15 s at 95°C, 60 s at 60°C, and 15 s at 95°C. Relative FCs for genes tested were calculated using the ΔΔCt method [33], normalized to GAPDH and then compared to LNCaP.

## Results

### Overlap of published marker gene sets

**Figure 1:**
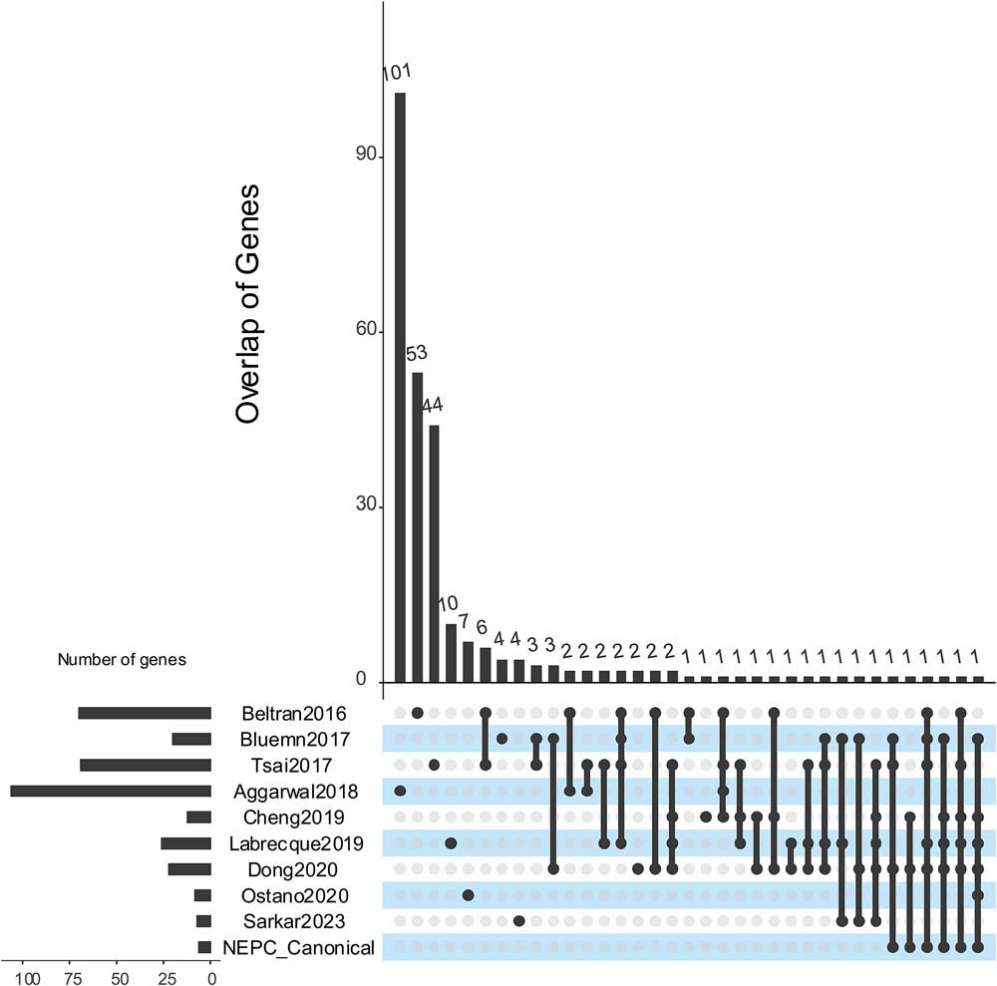
Overlap of NEPC marker genes published by selected literature.

We searched the literature for published NEPC marker gene sets mentioned in the introduction, namely, Beltran2016 [6], Tsai2017 [34], Bluemn2017 [35], Cheng2019 [36], Labrecque2019 [37], Dong2020 [20], Ostano2020 [38], and Sarkar2022 [39], in addition to six marker genes commonly used for clinical diagnose of NEPC, CHGA, PSA, NCAM1, ENO2, AR, and KLK3, named as NEPC canonical marker genes [11] (Fig. 1). The majority of genes within distinct marker sets tend to be unique or specific to a particular gene expression dataset employed for identification. The maximum pairwise overlap was observed for 11 genes between Beltran2016 and Tsai2017. Even the six canonical NEPC markers were rediscovered in certain marker gene sets but not universally across all of them. This lack of consensus in NEPC markers suggests the intricate nature of biological processes associated with NEPC progression and potential biases when simply comparing gene expression differences for different datasets.

### Integration of using knowledge in network identifies higher confidence neuroendocrine prostate cancer–related genes

**Figure 2:**
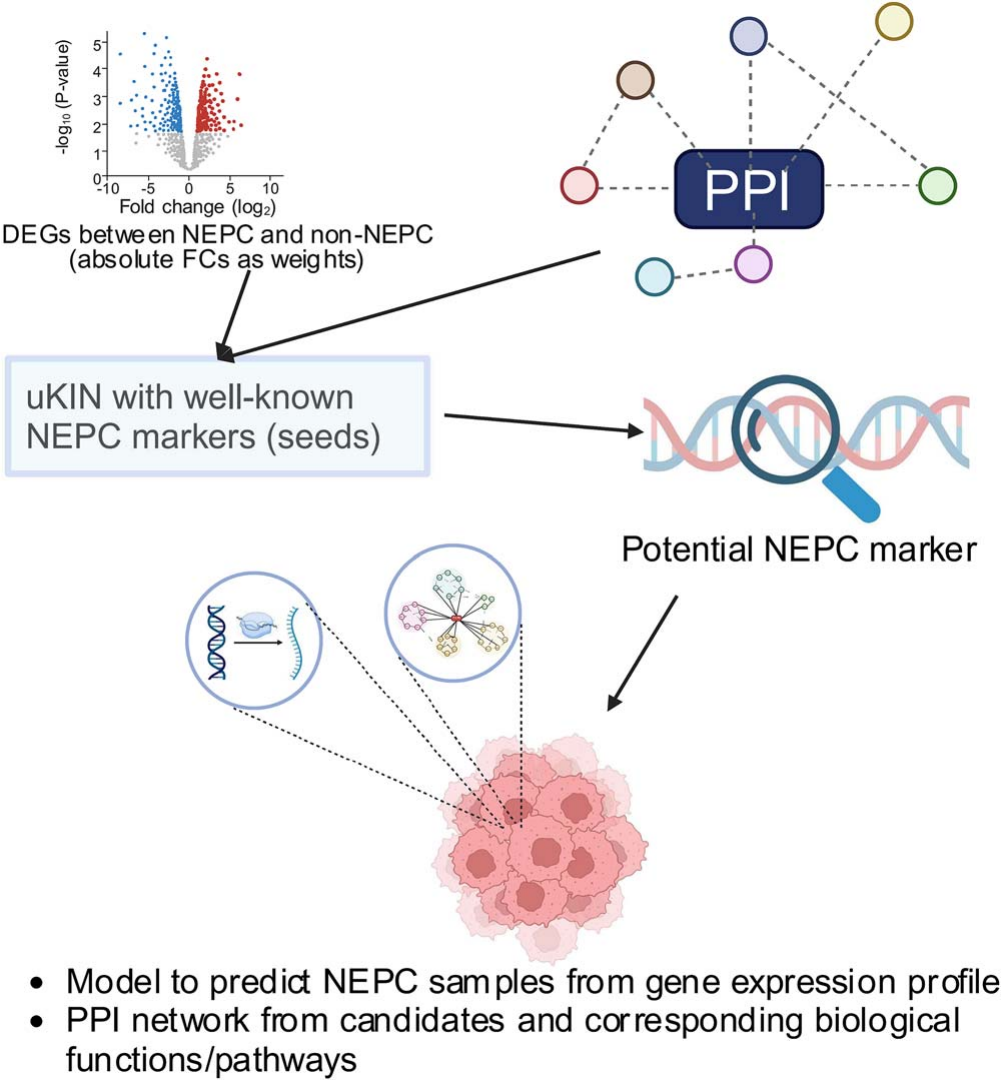
Flow chart of our approach.

To identify NEPC biomarkers combining different gene expression datasets while considering PPIs, we utilized uKIN to pinpoint potential biomarker genes (Fig. 2). Differential analysis was performed between 15 NEPC and 24 non-NEPC samples (NEPC_WCM_2016 dataset) and between 22 NEPC and 210 non-NEPC samples (PRAD_SU2C_2019 dataset), respectively, resulting in 729 and 894 DEGs based on cutoffs of FDR < 0.05 and |log_2_FC| >1 for NEPC_WCM_2016 and PRAD_SU2C_2019 dataset, respectively, Subsequently, we utilized uKIN, initiating from the six canonical NEPC markers as seed nodes, supported by PPI networks. We incorporated the average values of |log_2_FC| of gene expression FCs between NEPC and non-NEPC from both NEPC_WCM_2016 and PRAD_SU2C_2019 datasets as weights to enrich the new information in the network to guide the random walk and then rank genes according to their NEPC relatedness. A ranked list of genes along with their NEPC association scores was provided by uKIN. The top genes from the uKIN were considered potential NEPC biomarkers (Supplementary Fig. S1). Next, we derived a model based on selected top genes and further performed a functional analysis of top genes from the uKIN.

### Selection of top performing gene sets

To assess the effectiveness of identified top potential biomarker genes from ranked uKIN result in predicting the NEPC phenotype, we applied elastic net logistic regression to different numbers of top ranked genes identified by our approach, including top 10, 20, 30, and up to top 100 genes, respectively, aiming to select the optimal parameters that yield a highly regularized model with elastic net penalty. The 10-fold cross-validation and 10 repetitions were taken using the gene expression profiles in the training set to ensure that the cross-validated error falls within one standard error of the minimum. We also conducted analysis using Random Forests (RFs). However, the overall performance of RFs was not good as those by the logistic regression. Given the primary focus of this study is on NEPC biomarker identification, and the performance of logistic regression is sufficiently high, we did not perform more detailed comparisons with other algorithms.

**Figure 3:**
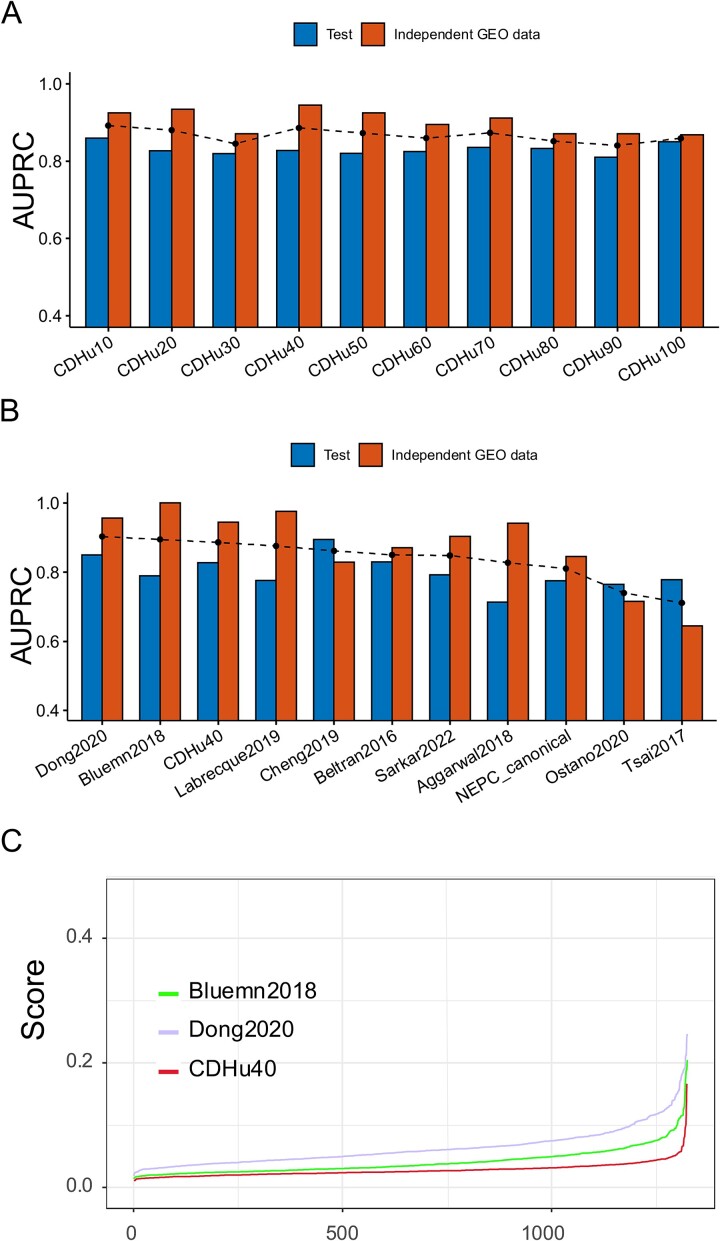
Performance of CDHu40 and other published NEPC marker gene sets. (A) AUPRC for top uKIN genes from 10 (CDHu10) up to 100 (CDHu100), where the dashed dot line is average values of AUPRC for two datasets. (B) Bar plot of AUPRC scores for each gene set. Gene sets were sorted by the average values (dashed dot line) of AUPRC. (C) NEPC scores of PRAD-TCGA samples estimated by Bluemn2018, Dong2020, and CDHu40, respectively.

Taking into account the balance between the quantity of marker genes involved and their performance (Fig. 3A), we chose the top 40 candidate genes, namely, CDHu40, as the gene set for NEPC biomarkers. The CDHu40 outperformed most of other NEPC marker gene sets listed here, except Bluemn2018 and Dong2020 (Fig. 3B), based on AUPRC scores on both test datasets and other independent GEO datasets collected in the study. Supplementary Tables S4 and S5 available online at http://bib.oxfordjournals.org/ listed other performance metrics on test and independent GEO datasets.

The CDHu40 score was defined as the NEPC prediction probability using the elastic net logistic regression model. Additionally, we computed NEPC scores using two other marker gene sets, Bluemn2018 and Dong2020, respectively, since both also exhibited superior performance on two test sets in the study. We calculated these scores utilizing the gene expression data of PRAD_TCGA (Fig. 3C). In general, CDHu40 scores were notably lower compared to the scores generated based on Dong2020 and Bluemn2018 gene sets. Some samples exhibited higher scores according to Dong2020. Given that the PRAD_TCGA samples were from primary PCa tumors that lack an NE phenotype, this suggests that CDHu40 provides a more accurate representation of the NEPC phenotype compared to Dong2020 and Bluemn2018, particularly in terms of minimizing false positive rates.

**Figure 4:**
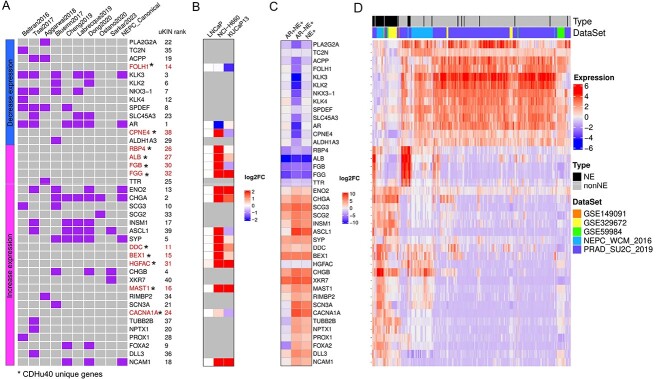
CDHu40 genes identified. (A) Overlap of CDHu40 and other marker gene sets in the literature. The left bar shows that marker genes were either down- or up- regulated in NEPC samples as we identified. Genes marked by asterisks were absent from all other published marker genes compared here. (B) qPRC validation on 17 selected marker genes in three PCa cell lines, LNCaP, NCI-H660, and KUCaP13. (C) Validation of gene expression changes (log2FC) by newly published RNA-seq tumor samples with AR and NE features, including both AR+ and AR− samples. (D) Expression profile of CDHu40 genes obtained by different data sets.

Except genes of CDHu40 recovered by other marker gene sets previously mentioned (Fig. 4A), more than a quarter (11 out of 40) of CDHu40 genes were absent from any of these sets (highlighted in red in Fig. 4A), although some of them have been investigated in independent studies. For instance, DDC was reported as a neuroendocrine marker in various human tumors originating from NE cells [40]. Prostate-specific membrane antigen (PSMA), often overexpressed in most prostate adenocarcinoma (AdPC) cells, serves as a marker for PC and becomes a target for molecular imaging. The down-regulation of the PSMA gene (FOLH1) in NEPC samples rendered it a marker capable of distinguishing NEPC from AdPC, and this suppressed expression of the PSMA gene in NEPC results in the failure of NEPC identification using PSMA-targeting imaging [41]. BEX1 was recognized for its involvement in the tumorigenesis of NE-specific tumors [42–44]. MAST1 played a role in modulating neuronal differentiation and cell cycle exit through P27 in neuroblastoma cells [45]. Notably, mutations in CACNA1A were linked to neuroendocrine dysregulation. [46]. ALB was identified as an independent risk factor for lymph node metastasis in gastric NE tumor patients [47]. FGB and FGG, both up-regulated in duodenopancreatic NE tumors (DPNETs), signified a dedifferentiation process in DPNET patients with poor outcomes [48]. Additionally, the fusion of CPNE4 and ACAD11 was identified in NE samples [49]. The qPCR experiments in three PCa cell lines, LNCaP, NCI-H660, and KUCaP13, were conducted to test the expression levels of these novel marker genes in addition to several well-known NEPC markers (Fig. 4B). The expression changes of most identified novel marker genes aligned with the predicted either up- or down-regulation in at least one of the two NEPC cell lines. Given newly published RNA-seq data for tumor samples [21], we validated the gene expression changes between NE^+^ and NE^−^ samples (Fig. 4C and Supplementary Fig. S2). Most CDHu40 genes showed consistent up- or down-regulation in AR^−^NE^+^ samples as predicted. These results collectively indicate that many unique genes in CDHu40 are strongly associated with the NEPC phenotype and warrant further in-depth understanding and investigations.

Figure 4D showcases the expression patterns of these CDHu40 genes by two-way clustering across various independent datasets with documented NEPC information that were generated by independent groups and collected for our study. Discernible are two major groups of genes, either down-regulated in NEPC samples, exemplified by AR, KLK3, FOLH1, etc., or up-regulated in NEPC samples, e.g. CHGA, ENO2, SYP, DDC, BEX1, HGFAC, and others. However, several subsets of non-NEPC samples were observed with higher expressions of RBP4, ALB, FGB, FGG, and TTR or DDC, BEX1, HGFAC, and CHGB (Fig. 4D), which typically show augmented expression levels in the majority of NEPC samples. It suggests potential subtypes of certain documented non-NEPC samples that might come with some NEPC features or were progressing toward NEPC. Such information may bring new insights into the molecular mechanisms of the development of NE phenotype from PCa.

### Validation of CDHu40 score in multiple prostate cancer datasets

**Figure 5:**
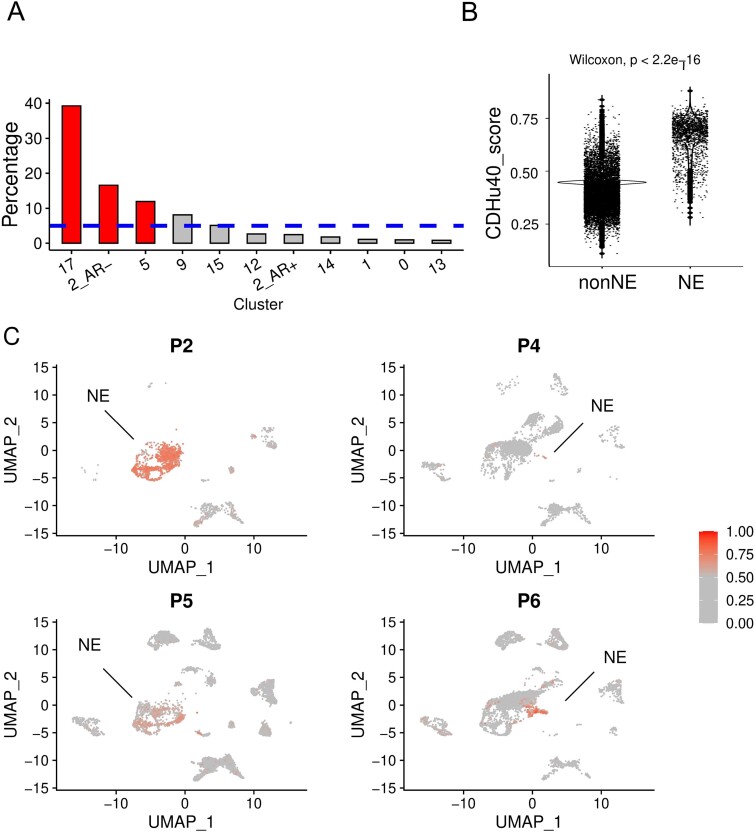
Association between CDHu40 score and NEPC cells from scRNA-seq datasets. (A) Percentage of cells with higher CDHu40 scores in each cluster recognized by Asberry *et al.* [19] at Day 14 after NED. The bars represent the significantly (*P* < .05) higher ratio, whereas the dashed line is the average percentage of high-CDHu40 score in all cells in the sample. (B) CDHu40 scores for NE cells and non-NE cells. (C) Cells with higher CDHu40 scores were marked given the patient samples with the scRNA-seq by Dong *et al*. [20].

We re-analyzed two sets of previously published scRNA-seq data [19,20] and subsequently applied the CDHu40 score at the single cell level to distinguish NEPC cells from others. Significant enrichments of cells with higher CDHu40 scores were observed in clusters 5 and 17, as well as AR− cells in cluster 2 (Fig. 5A) at Day 14 subjected to ENZ treatment inducing neuroendocrine differentiation (NED), consistent with the results reported and the phenotype changes observed in the experiments [19].

Dong *et al*. [20] published scRNA-seq datasets based on samples from CRPC patients characterizing the tumor cell diversity in 2020. Among these patients, four were clinically determined to have undergone NED. NE cells showed significantly higher CDHu40 scores compared to non-NE cells (Fig. 5B and C), underscoring the robustness of CDHu40 score and strong association of CDHu40 score with the NEPC phenotype even at the single cell level.

### Strong correlation between CDHu40 and survival data

**Figure 6:**
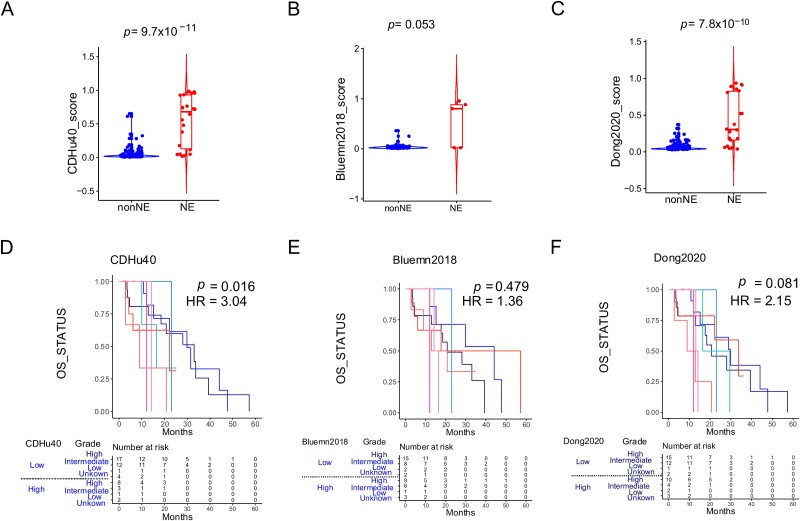
Examination of CDHu40 score on PRAD_SU2C_2019 samples. NEPC scores on clinical NE and non-NE samples were calculated by (A) CDHu40, (B) Bluemn2018, and (C) Dong2020. Corresponding survival differences were evaluated using stratified Cox regression with the pathological grade being adjusted as a stratification factor according to scores by (D) CDHu40, (E) Bluemn2018, and (F) Dong2020, respectively. HR: hazard ratio of high score samples.

Taking the dataset of PRAD_SU2C_2019 [14] as another example, we evaluated scores estimated by the CDHu40, Dong2020, and Bluemn2018, respectively, across all NE and non-NE samples (Fig. 6A–C) to examine these scores with the clinical diagnoses. CDHu40 scores displayed a significant increase in NE samples in comparison to non-NE samples, aligning consistently with the NE phenotype diagnosed by clinicians. More strikingly, by using CDHu40 as a criterion, the higher CDHu40 score group reports much worse OS compared with the low CDHu40 score group, with a hazard ratio (HR) = 3.04 and a statistically significant *P*-value, *P* = .016 (Fig. 6D). The results are calculated using stratified Cox regression with pathological grade being adjusted as a stratification factor. However, no notable difference was observed for patients classified by higher or lower scores based on Bluemn2018 and Dong2020 (Fig. 6E and F) or generated by other marker sets (Supplementary Fig. S3).

Similar trends were evident when we tested the PRAD-TCGA datasets (Supplementary Fig. S4). Patients with augmented CDHu40 scores encountered significantly (log rank test *P* = 3.0 × 10^−4^) shorter disease-free survival times than those with lower CDHu40 scores (Supplementary Fig. S4A). No remarkable differences were noted between higher and lower scores identified by either Bluemn2018 (Supplementary Fig. S4B) or Dong2020 (Supplementary Fig. S4C). These findings indicate the potential of the CDHu40 score as a promising prognostic indicator of patients with the NE phenotype. Samples lacking the NE phenotype but exhibiting higher CDHu40 score can be inferred to be at risk of developing the NE phenotype based on their CDHu40 scores.

### Functional analysis on top 500 candidate genes

**Figure 7:**
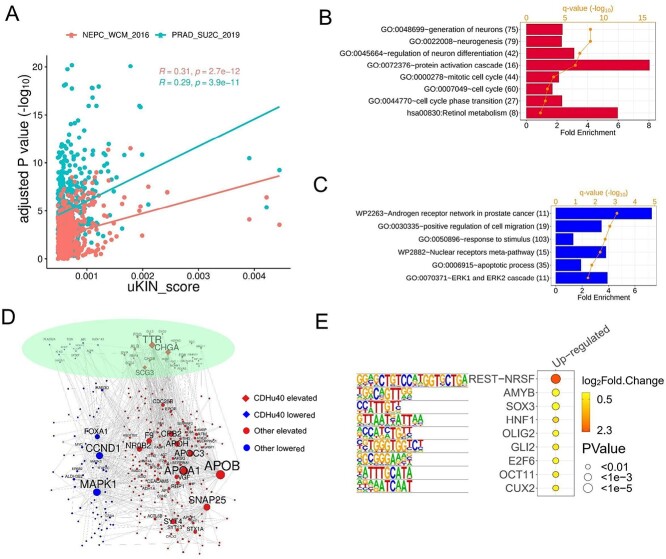
Top 500 genes identified by our methods. (A) Correlation between uKIN scores of top 500 gene and corresponding adjusted *P*-values (−log10) determined by differential analysis on two datasets, NEPC_WCM_2016 and PRAD_SU2C_2019, respectively. Gene ontology and Kyoto Encyclopedia of Genes and Genomes (KEGG) pathway enriched in (B) up-regulated genes and (C) down-regulated genes. (D) PPI network of top 500 genes including. CDHu40 maerker genes and other genes with elevated and lower expression levels, respectively, in NEPC samples. (E) Motifs enriched in the regions from upstream (2 kb) to downstream (500 bp) of 330 up-regulated candidate genes.

We ranked DEGs by adjusted *P*-value for two datasets, NEPC_WCM_2016 and PRAD_SU2C_2019, respectively, and then compared them with the top 500 genes identified by uKIN. Two hundred twenty-two and 218 out of top 500 uKIN marker genes (44.4% and 43.6%) were also top 500 DEGs for PRAD_SU2C_2019 and NEPC_WCM_2016, respectively. However, the correlations between the uKIN scores and adjusted *P*-values (in −log_10_ scale) were not strong (Fig. 7A), indicating that the additional information from the PPI network significantly influences the final ranking of selected genes based on the uKIN model.

The biologically functional enrichment analysis was conducted by using DAVID [30,31] on the genes that were up- and down-regulated, respectively, in NEPC samples among the top 500 candidates identified in the study. The analysis revealed some biological processes significantly enriched in up-regulated genes, such as the generation of neurons, neurogenesis, regulation of neuron differentiation, and cell cycle (Fig. 7B), in accordance with NE features observed. Interestingly, genes activated in NEPC samples enriched for protein activation cascade may suggest their potential contributions to neurogenesis or cell cycle processes.

Conversely, an enrichment of the AR network in PCa was observed in down-regulated genes (Fig. 7C), corresponding to the inhibition of AR expressions in NE samples. Additionally, apoptotic processes and cell migration were enriched in down-regulated genes, suggesting that repressed cell migration might affect or even lead to a shift toward a more vital NE status.

Figure 7D exhibits the PPI network for the top 500 candidate genes. Among 40 CDHu40 genes, 11 genes serve as hub genes, being connected with at least five other genes in the network. This indicates the importance of CDHu40 in establishing connections among genes, leading to the featured functions of NEPC. Other hub genes such as CCND1 [50] and CDC25B [51] are associated with the cell cycle. CDC25B induces cellular senescence and correlates with tumor suppression in a p53-dependent manner. Additionally, APOA1 was identified to be upregulated in normal PC. Augmented APOA1 reflects its potential role in driving therapeutic resistance and disease progression by reprogramming the lipid metabolic network of tumor cells [52]; APOB, APOC3, and APOH may function like APOA1. Other highlighted gene was SYT4, which is a well-characterized marker for NE tumors, [53,54]. NR0B2 is a novel androgen receptor co-repressor in mouse Sertoli cells [55]. MAPK1 plays a role in the activation of Erk1/2-mitogen-activated protein kinases (MAPK) signal transduction pathway in SCLC [56], which has NE features. FOXA1 inhibits prostate cancer NED [57].

We investigated the consensus sequences in the region spanning upstream 2 kb to downstream 500 bp of up-regulated genes and down-regulated genes, respectively, in the top 500 gene candidates (Fig. 7E). The repressor element-1 (RE-1) silencing transcription factor (REST) motif was observed to be enriched in the up-regulated genes, including targets CHGA, CHGB, and SYP based on the motif analysis. The known functions of REST involve the repression of neural genes and the negative regulation of neurogenesis. Up-regulation of REST target genes in NEPC samples indicates a weakening or absence of the repression role of REST, in turn, activating or contributing to the NE features. Another noteworthy finding is the involvement of E2F6, a member of the E2F family, activated in the NEPC samples. E2F6 plays a critical role during the G1/S transition in the mammalian cell cycle. This suggests potential dysfunction of the cell cycle in NEPC through the activity of E2F6 and its target genes. Interestingly, no significantly enriched motifs were observed in down-regulated genes among the top 500 candidates.

## Conclusions and discussions

PCa stands as the most prevalent cancer among men in the USA [1]. Hormone treatment is the frontline treatment regimen for PCa patients due to the disease sensitivity towards androgen. All existing therapies for PCa, particularly the next-generation ASI drugs, lead to the development of a particular subtype of PCa that exhibits the NE-like phenotype that is no longer responsive to any type of antiandrogen treatment [58]. Not only is NEPC treatment-resistant, but its complex genetic heterogeneity also contributes to misdiagnosis and challenges in recognition.

Due to the lack of biopsy samples of metastatic NEPC, poor characterization of the disease is still prevalent [9]. One of the hurdles of disease identification is the consequent undersampling of mixed histology of the NEPC samples. Genotypic and phenotypic evaluation only represents small lesions of the actual diverse genetic profile of the disease. Several studies have shown potential diagnosis markers for NEPC such as CGA, NCAM1, SYP, and NSE; however, their expressions do not always coincide among patients. To address this biopsy barrier, we established potential diagnostic and prognostic markers for NEPC patients.

Here we proposed a novel integration method incorporating differential gene expression analysis between NEPC and non-NEPC samples as well as the uKIN algorithm based on the PPI network starting with several well-known NEPC biomarkers. The approach effectively generates a list of candidate biomarker genes for NEPC. Our analysis of the top 500 candidates revealed enrichment in neural-related features and cell cycle process enriched in genes up-regulated in NEPC, along with repression in the AR network in NEPC. The PPI network for these top 500 genes identified hub genes associated with the cell cycle and progression of NED. Additionally, motifs of REST and E2F6 were enriched in promoter regions of these top candidates, suggesting their involvement in the generation of NE features and cell cycle regulation.

We specifically selected the top 40 candidate genes, termed CDHu40, which includes some different targets not reported in other NEPC marker sets. The CDHu40 gene set exhibited functional relations with NE features, providing further insights into the underlying NED mechanisms. Particularly, CDHu40 genes demonstrated robust and efficient performance in predicting NEPC samples using both bulk mRNA expression and single cell expression data compared with other published marker gene sets. In this paper, we used the AUPRC, the golden-standard and most popular statistical tool, as the overall criteria to evaluate the precision and recall across a range of different threshold values. An alternative approach is to consider the F-scores, which may be more suitable for imbalanced data. Further research in this area is warranted. More importantly, the CDHu40 score emerges as a better diagnostic marker for NEPC and a reliable prognostic marker for NEPC patients.

Nevertheless, due to the heterogeneity of NEPC, most of the time, the identified markers will not reflect the clinical identification of NEPC/NE-CRPC. For example, variations in CDHu40 gene expression profiles were observed across diverse datasets (Fig. 4D). Notably, distinct subsets of non-NEPC samples were noted with elevated expressions of either RBP4, ALB, FGB, FGG, and TTR, or DDC, BEX1, HGFAC, and CHGB (Fig. 4D), which typically exhibit heightened expression levels in the majority of NEPC samples. This observation suggests potential subtypes within documented non-NEPC samples that may harbor some NEPC features or could be progressing toward NEPC. Collectively, these insights contribute to improving diagnosis and imaging options for NEPC patients.

**Key Points**

- Our study integrates gene expression variances in multiple neuroendocrine prostate cancer (NEPC) studies and protein–protein interaction (PPI) network to pinpoint a specific set of NEPC maker genes, namely, CDHu40. With PPI networks, we identify genes that can distinguish a specific cancer phenotype and predict clinical prognostic significance. This approach may offer additional insights for similar analyses in other cancer types.
- Our paper holds significant translational importance due to the current lack of suitable unique identification markers for NEPC. The CDHu40 genes demonstrated strong and efficient performance in predicting NEPC samples using both bulk messenger RNA expression and single-cell expression data. Notably, the CDHu40 score proves to be a superior diagnostic marker for NEPC and a reliable prognostic marker for NEPC patients compared to other published marker gene sets. The CDHu40 score may have substantial translational relevance based on the results presented in the paper.
- The PPI network analysis of the top 500 candidate marker genes, identified through our innovative approach, revealed hub genes associated with the cell cycle and neuroendocrine differentiation progression. Additionally, the promoter regions of these top candidates showed enriched motifs of REST and E2F6, suggesting their potential role in shaping neuroendocrine characteristics and regulating the cell cycle. These findings offer deeper insights into NEPC mechanisms.

## Supplementary Material

SuppFigure1_bbae471

SuppFigure2_bbae471

SuppFigure3_bbae471

SuppFigure4_bbae471

CDHu40_BIB_v6_SuppTab_bbae471

## Data Availability

The authors declare that the data supporting the findings and conclusions of this study are available within the paper and its Supplementary Information file. Other data are available from the corresponding author upon reasonable requests.
