# Supplementary figures and images for: CDHu40: a novel marker gene set of neuroendocrine prostate cancer
Source: Brief Bioinform. 2024 Sep 25;25(6):bbae471. doi: 10.1093/bib/bbae471 (PMC11422505; doi:10.1093/bib/bbae471)

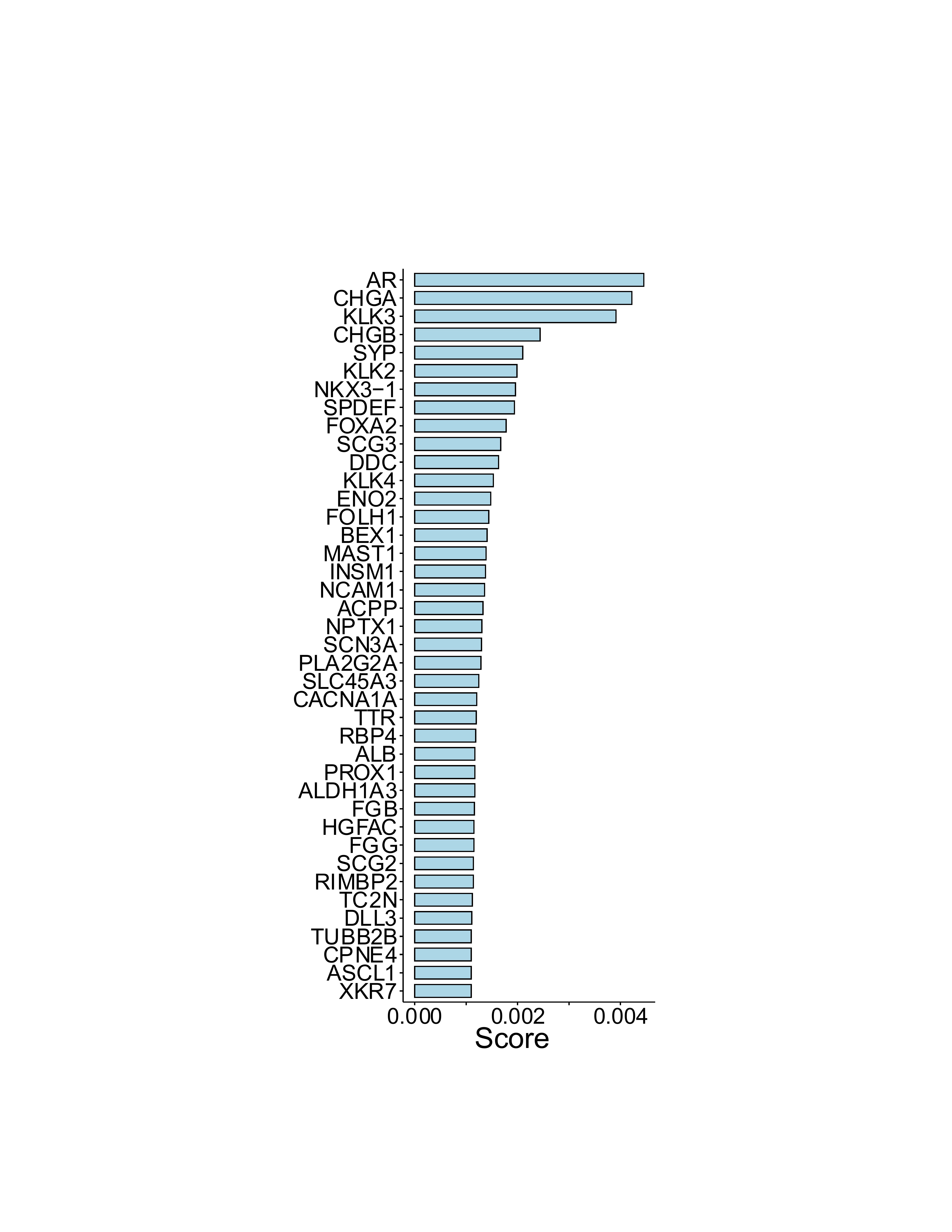

Supplement: SuppFigure1_bbae471 [file suppfigure1_bbae471.jpeg]

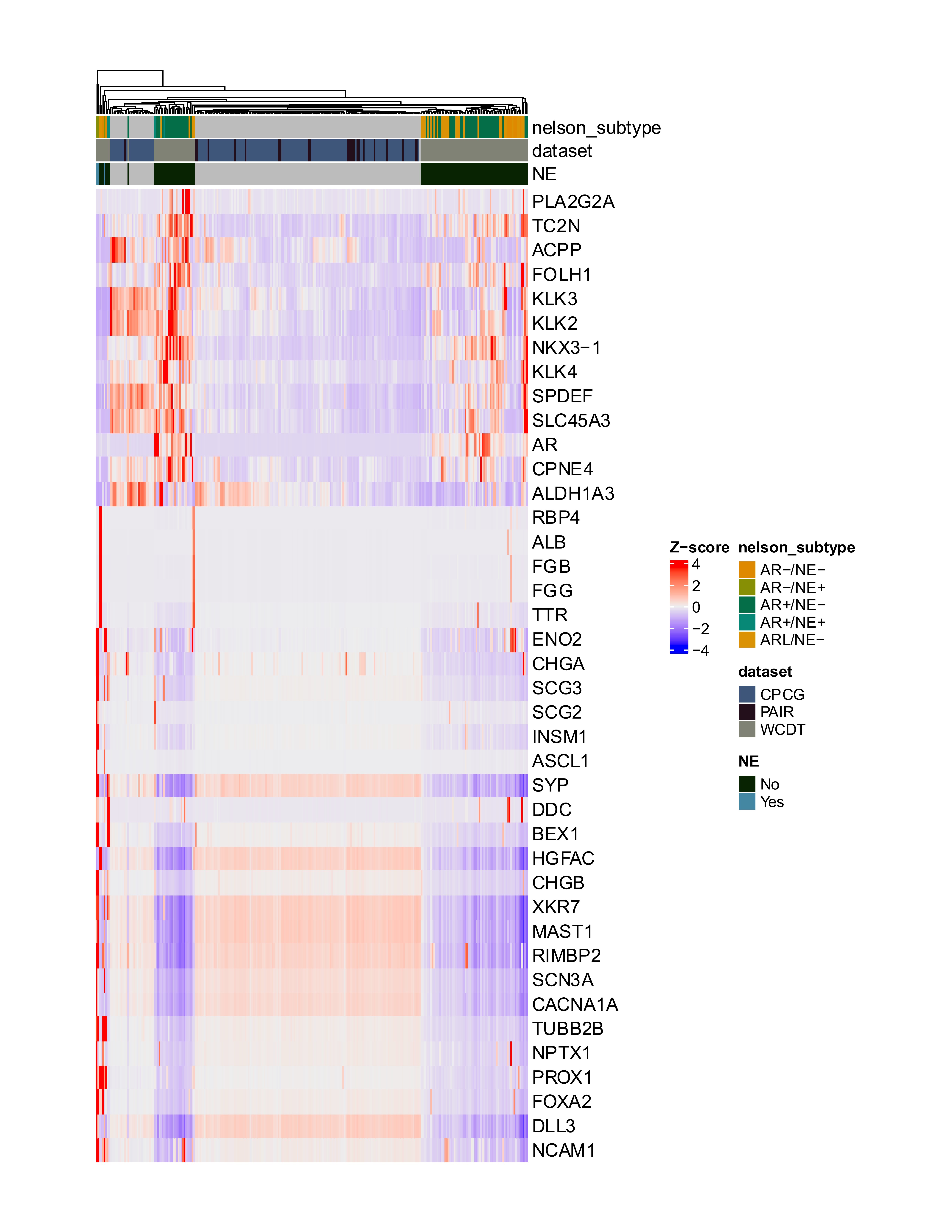

Supplement: SuppFigure2_bbae471 [file suppfigure2_bbae471.jpeg]

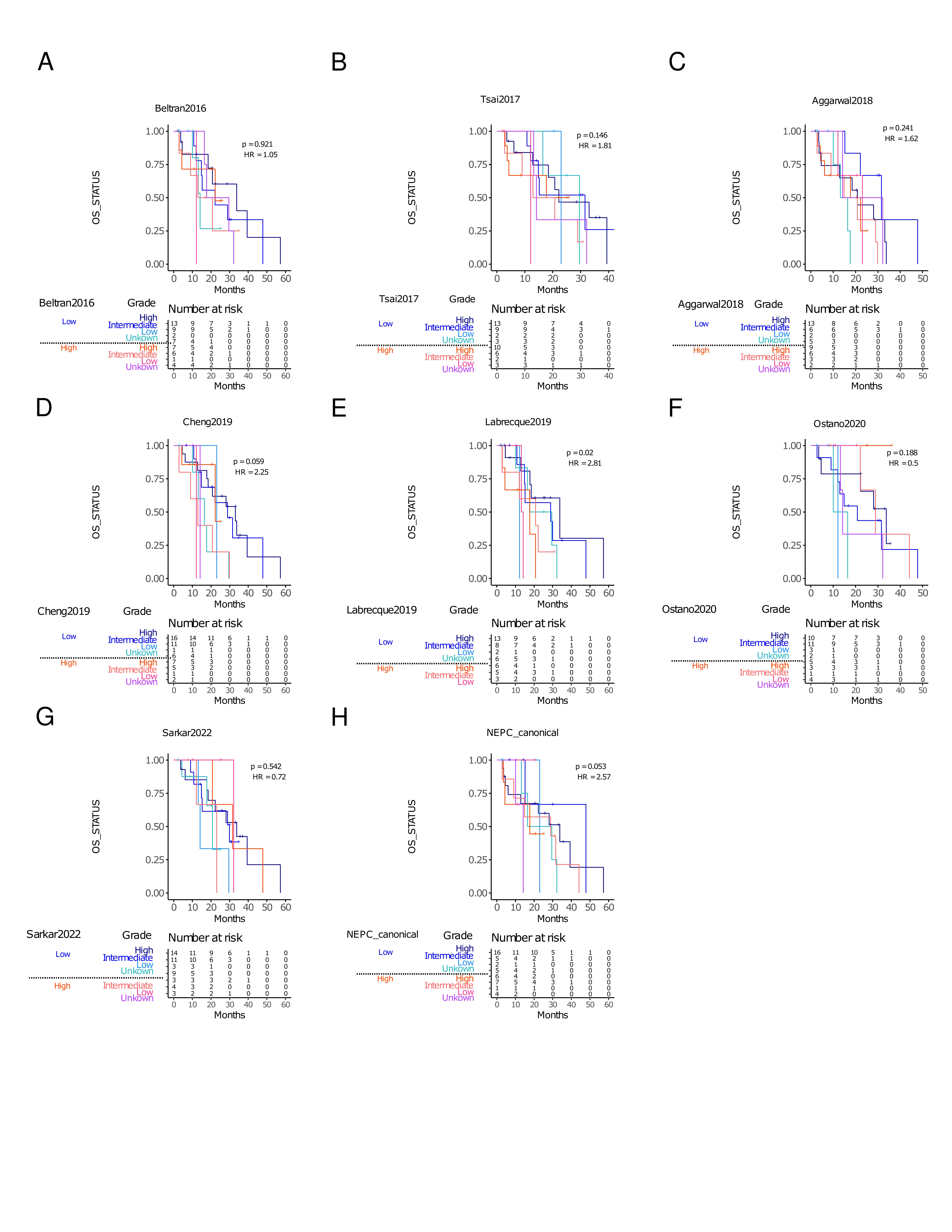

Supplement: SuppFigure3_bbae471 [file suppfigure3_bbae471.jpeg]

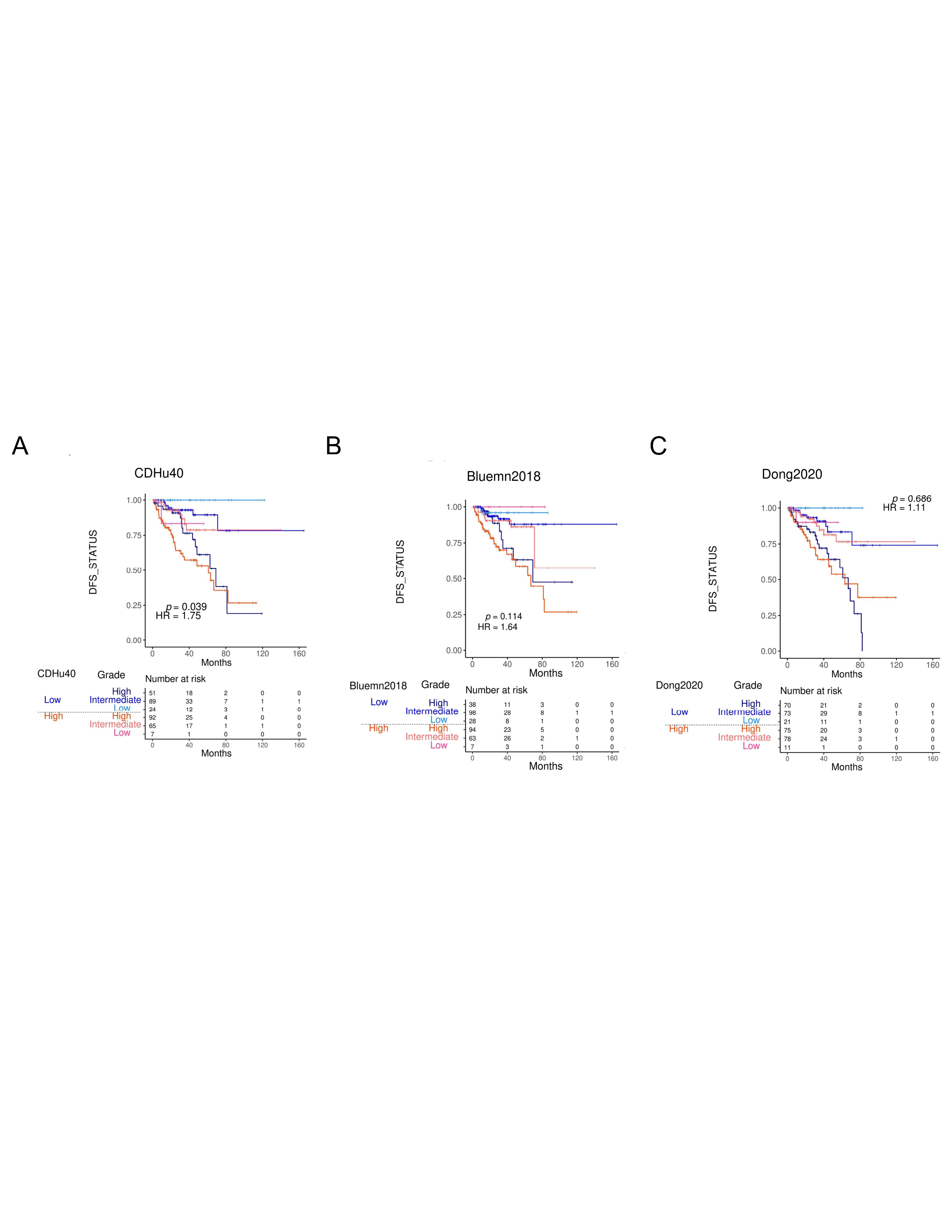

Supplement: SuppFigure4_bbae471 [file suppfigure4_bbae471.jpeg]
