# Supplementary material for: CDHu40: a novel marker gene set of neuroendocrine prostate cancer
Source: Brief Bioinform. 2024 Sep 25;25(6):bbae471. doi: 10.1093/bib/bbae471 (PMC11422505; doi:10.1093/bib/bbae471)
Supplement: CDHu40_BIB_v6_SuppTab_bbae471 [file cdhu40_bib_v6_supptab_bbae471.docx]

1. **Supplementary Tables**

**Table S1. NEPC Marker gene sets in selected literatures.**

| Gene set | No. of genes | | Description | Reference (PMID) |
| --- | --- | --- | --- | --- |
| Beltran2016[1] | | 70 | Integration of copy number variation, methylation, and gene expression profiles | PMID: 26855148 |
| Bluemn2017[2] | | 20 | 10-gene signature concordant with Beltran et al. in 2016 and Hieronymus et al together with 10 genes low expression in NEPC | PMID: 29017058 |
| Tsai2017[3] | | 69 | Estimated gene expressions based on microarray data for NEPC and adenocarcinoma samples where genes with consistent outlier expression relative to adenocarcinomas were identified as NEPC markers | PMID: 29132337 |
| Aggarwal2018[4] | | 106 | t-SCNC gene expression signature distinguishes small-cell PCa and normal PCa | PMID: 29985747 |
| Cheng2019[5] | | 12 | Based on literature and gene expression profiles from data generated by Beltran et al. | PMID: 31763364 |
| Labrecque2019[6] | | 26 | combined NE-associated genes in 2019 which were discovered by Zhang et al. in 2015 [PMID: 26071481], Mu et al. in 2017 [PMID: 28059768], Bishop et al in 2017 [PMID: 27784708] in addition to AR associated genes selected from the literature [Tan et al 2012. PMID: 22083957, Cheng et al. 2003 PMID: 12865322, Rickman et al 2009 PMID: 19293179] to classify mCRPC phenotypes | PMID: 31361600 |
| Dong2020[7] | | 22 | Used 14 NE genes and 8 AR-related genes to score the cells to locate NE cells | PMID: 33328604 |
| Ostano2020[8] | | 8 | adopted 8 genes in 2020 to classify NEPC and normal PC by penalized logistic regression which achieved a high AUC score (>0.87) for the datasets by Beltran in 2016 and GSE66187 [[9] PMID: 26071481]. | PMID: 32041153 |
| Sarkar2023[10] | | 7 | compared nucleosome phasing scores (NPS) between NE samples and AR-positive adenocarcinoma (ARPC) in 2022 where 7 genes showed significant expression differences between NEPC and ARPC with p values less than 0.05. | PMID: 36399432 |

**Table S2: Summary of metadata for PRAD_SU2C_2019 data.**

| SEX | All are males |
| --- | --- |
| AGE_AT_DIAGNOSIS | 38.6 - 89.0 |
| GLEASON_SCORE | 6:n=27, 7:n=103, 8:n=67, 9:n=122, 10:n=24, 11:n=1, Unknown: n=84 |
| Grade | Low: n=27, Intermediate: n=103, High: n=214, Unknown: n=84 |

**Table S3: Summary of metadata for PRAD_TCGA data.**

| SEX | All are males |
| --- | --- |
| AGE | 41-79 |
| GLEASON_SCORE | 6:n=45, 7:n=250, 8:n=64, 9:n=137, 10:n=4 |
| Grade | Low: n=45, Intermediate: n=250, High: n=205 |
| Stage | T1:n=178, T2:n=173, T3:n=53, T4:n=2, Unknown:n=94 |

**Table S4: Model performance for Test data including sensitivity, specificity, accuracy, PPV, NPV, and AUPRC.**

|  | Threshold | sensitivity | specificity | accuracy | PPV* | NPV* | AUPRC* |
| --- | --- | --- | --- | --- | --- | --- | --- |
| Beltran2016 | 0.25 | 0.6667 | 0.9836 | 0.9429 | 0.8571 | 0.9524 | 0.8293 |
| Tsai2017 |  | 0.6667 | 0.9344 | 0.9000 | 0.6000 | 0.9500 | 0.7781 |
| Aggarwal2018 |  | 0.5556 | 0.9672 | 0.9143 | 0.7143 | 0.9365 | 0.7128 |
| Bluemn2018 |  | 0.7778 | 0.9508 | 0.9286 | 0.7000 | 0.9667 | 0.7890 |
| Cheng2019 |  | 0.7778 | 0.9672 | 0.9429 | 0.7778 | 0.9672 | 0.8941 |
| Labrecque2019 |  | 0.6667 | 0.9836 | 0.9429 | 0.8571 | 0.9524 | 0.7756 |
| Dong2020 |  | 0.5556 | 1.0000 | 0.9429 | 1.0000 | 0.9385 | 0.8497 |
| Ostano2020 |  | 0.6667 | 0.9836 | 0.9429 | 0.8571 | 0.9524 | 0.7646 |
| Sarkar2022 |  | 0.6667 | 0.9672 | 0.9286 | 0.7500 | 0.9516 | 0.7921 |
| NEPC_canonical |  | 0.6667 | 0.9344 | 0.9000 | 0.6000 | 0.9500 | 0.7750 |
| CDHu40 |  | 0.6667 | 0.9836 | 0.9429 | 0.8571 | 0.9524 | 0.8275 |
| Beltran2016 | 0.5 | 0.4444 | 1.0000 | 0.9286 | 1.0000 | 0.9242 | 0.8293 |
| Tsai2017 |  | 0.6667 | 0.9672 | 0.9286 | 0.7500 | 0.9516 | 0.7781 |
| Aggarwal2018 |  | 0.3333 | 1.0000 | 0.9143 | 1.0000 | 0.9104 | 0.7128 |
| Bluemn2018 |  | 0.6667 | 1.0000 | 0.9571 | 1.0000 | 0.9531 | 0.7890 |
| Cheng2019 |  | 0.5556 | 1.0000 | 0.9429 | 1.0000 | 0.9385 | 0.8941 |
| Labrecque2019 |  | 0.6667 | 1.0000 | 0.9571 | 1.0000 | 0.9531 | 0.7756 |
| Dong2020 |  | 0.5556 | 1.0000 | 0.9429 | 1.0000 | 0.9385 | 0.8497 |
| Ostano2020 |  | 0.4444 | 1.0000 | 0.9286 | 1.0000 | 0.9242 | 0.7646 |
| Sarkar2022 |  | 0.6667 | 1.0000 | 0.9571 | 1.0000 | 0.9531 | 0.7921 |
| NEPC_canonical |  | 0.6667 | 1.0000 | 0.9571 | 1.0000 | 0.9531 | 0.7750 |
| CDHu40 |  | 0.6667 | 1.0000 | 0.9571 | 1.0000 | 0.9531 | 0.8275 |
| Beltran2016 | 0.75 | 0.2222 | 1.0000 | 0.9000 | 1.0000 | 0.8971 | 0.8293 |
| Tsai2017 |  | 0.6667 | 0.9836 | 0.9429 | 0.8571 | 0.9524 | 0.7781 |
| Aggarwal2018 |  | 0.2222 | 1.0000 | 0.9000 | 1.0000 | 0.8971 | 0.7128 |
| Bluemn2018 |  | 0.6667 | 1.0000 | 0.9571 | 1.0000 | 0.9531 | 0.7890 |
| Cheng2019 |  | 0.5556 | 1.0000 | 0.9429 | 1.0000 | 0.9385 | 0.8941 |
| Labrecque2019 |  | 0.5556 | 1.0000 | 0.9429 | 1.0000 | 0.9385 | 0.7756 |
| Dong2020 |  | 0.5556 | 1.0000 | 0.9429 | 1.0000 | 0.9385 | 0.8497 |
| Ostano2020 |  | 0.0000 | 1.0000 | 0.8714 | N/A | 0.8714 | 0.7646 |
| Sarkar2022 |  | 0.3333 | 1.0000 | 0.9143 | 1.0000 | 0.9104 | 0.7921 |
| NEPC_canonical |  | 0.4444 | 1.0000 | 0.9286 | 1.0000 | 0.9242 | 0.7750 |
| CDHu40 |  | 0.5556 | 1.0000 | 0.9429 | 1.0000 | 0.9385 | 0.8275 |

* PPV: positive predictive value; NPV: negative predictive value; AUPRC: area under precision-recall curve.

**Table S5: Model performance based on independent GEO datasets.**

|  | Threshold | sensitivity | specificity | accuracy | PPV* | NPV* | AUPRC* |
| --- | --- | --- | --- | --- | --- | --- | --- |
| Beltran2016 | 0.25 | 1.0000 | 0.9130 | 0.9500 | 0.8947 | 1.0000 | 0.8708 |
| Tsai2017 |  | 0.6471 | 0.8696 | 0.7750 | 0.7857 | 0.7692 | 0.6440 |
| Aggarwal2018 |  | 0.7059 | 0.9565 | 0.8500 | 0.9231 | 0.8148 | 0.9414 |
| Bluemn2018 |  | 1.0000 | 0.8261 | 0.9000 | 0.8095 | 1.0000 | 1.0000 |
| Cheng2019 |  | 0.4118 | 0.8696 | 0.6750 | 0.7000 | 0.6667 | 0.8291 |
| Labrecque2019 |  | 1.0000 | 0.7826 | 0.8750 | 0.7727 | 1.0000 | 0.9761 |
| Dong2020 |  | 0.8824 | 0.9565 | 0.9250 | 0.9375 | 0.9167 | 0.9562 |
| Ostano2020 |  | 0.4118 | 0.8696 | 0.6750 | 0.7000 | 0.6667 | 0.7150 |
| Sarkar2022 |  | 1.0000 | 0.8696 | 0.9250 | 0.8500 | 1.0000 | 0.9039 |
| NEPC_canonical |  | 1.0000 | 0.8696 | 0.9250 | 0.8500 | 1.0000 | 0.8457 |
| CDHu40 |  | 1.0000 | 0.9565 | 0.9750 | 0.9444 | 1.0000 | 0.9444 |
| Beltran2016 | 0.5 | 1.0000 | 0.9565 | 0.9750 | 0.9444 | 1.0000 | 0.8708 |
| Tsai2017 |  | 0.6471 | 0.8696 | 0.7750 | 0.7857 | 0.7692 | 0.6440 |
| Aggarwal2018 |  | 0.1176 | 1.0000 | 0.6250 | 1.0000 | 0.6053 | 0.9414 |
| Bluemn2018 |  | 1.0000 | 0.9565 | 0.9750 | 0.9444 | 1.0000 | 1.0000 |
| Cheng2019 |  | 0.3529 | 0.8696 | 0.6500 | 0.6667 | 0.6452 | 0.8291 |
| Labrecque2019 |  | 1.0000 | 0.9565 | 0.9750 | 0.9444 | 1.0000 | 0.9761 |
| Dong2020 |  | 0.4706 | 1.0000 | 0.7750 | 1.0000 | 0.7188 | 0.9562 |
| Ostano2020 |  | 0.2941 | 1.0000 | 0.7000 | 1.0000 | 0.6571 | 0.7150 |
| Sarkar2022 |  | 0.7059 | 0.9130 | 0.8250 | 0.8571 | 0.8077 | 0.9039 |
| NEPC_canonical |  | 0.9412 | 0.9130 | 0.9250 | 0.8889 | 0.9545 | 0.8457 |
| CDHu40 |  | 0.8824 | 0.9565 | 0.9250 | 0.9375 | 0.9167 | 0.9444 |
| Beltran2016 | 0.75 | 0.2353 | 0.9565 | 0.6500 | 0.8000 | 0.6286 | 0.8708 |
| Tsai2017 |  | 0.6471 | 0.8696 | 0.7750 | 0.7857 | 0.7692 | 0.6440 |
| Aggarwal2018 |  | 0.0000 | 1.0000 | 0.5750 | N/A | 0.5750 | 0.9414 |
| Bluemn2018 |  | 0.7059 | 1.0000 | 0.8750 | 1.0000 | 0.8214 | 1.0000 |
| Cheng2019 |  | 0.3529 | 0.9565 | 0.7000 | 0.8571 | 0.6667 | 0.8291 |
| Labrecque2019 |  | 0.7647 | 0.9565 | 0.8750 | 0.9286 | 0.8462 | 0.9761 |
| Dong2020 |  | 0.2941 | 1.0000 | 0.7000 | 1.0000 | 0.6571 | 0.9562 |
| Ostano2020 |  | 0.0000 | 1.0000 | 0.5750 | N/A | 0.5750 | 0.7150 |
| Sarkar2022 |  | 0.6471 | 0.9565 | 0.8250 | 0.9167 | 0.7857 | 0.9039 |
| NEPC_canonical |  | 0.5294 | 0.9565 | 0.7750 | 0.9000 | 0.7333 | 0.8457 |
| CDHu40 |  | 0.3529 | 0.9565 | 0.7000 | 0.8571 | 0.6667 | 0.9444 |

* PPV: positive predictive value; NPV: negative predictive value; AUPRC: area under precision-recall curve.

**References**

1. Beltran H, Prandi D, Mosquera JM et al. Divergent clonal evolution of castration-resistant neuroendocrine prostate cancer, Nat Med 2016;22:298-305.

2. Bluemn EG, Coleman IM, Lucas JM et al. Androgen Receptor Pathway-Independent Prostate Cancer Is Sustained through FGF Signaling, Cancer Cell 2017;32:474-489 e476.

3. Tsai HK, Lehrer J, Alshalalfa M et al. Gene expression signatures of neuroendocrine prostate cancer and primary small cell prostatic carcinoma, BMC Cancer 2017;17:759.

4. Aggarwal R, Huang J, Alumkal JJ et al. Clinical and Genomic Characterization of Treatment-Emergent Small-Cell Neuroendocrine Prostate Cancer: A Multi-institutional Prospective Study, J Clin Oncol 2018;36:2492-2503.

5. Cheng S, Yu X. Bioinformatics analyses of publicly available NEPCa datasets, Am J Clin Exp Urol 2019;7:327-340.

6. Labrecque MP, Coleman IM, Brown LG et al. Molecular profiling stratifies diverse phenotypes of treatment-refractory metastatic castration-resistant prostate cancer, J Clin Invest 2019;129:4492-4505.

7. Dong B, Miao J, Wang Y et al. Single-cell analysis supports a luminal-neuroendocrine transdifferentiation in human prostate cancer, Commun Biol 2020;3:778.

8. Ostano P, Mello-Grand M, Sesia D et al. Gene Expression Signature Predictive of Neuroendocrine Transformation in Prostate Adenocarcinoma, Int J Mol Sci 2020;21.

9. Zhang X, Coleman IM, Brown LG et al. SRRM4 Expression and the Loss of REST Activity May Promote the Emergence of the Neuroendocrine Phenotype in Castration-Resistant Prostate Cancer, Clin Cancer Res 2015;21:4698-4708.

10. De Sarkar N, Patton RD, Doebley AL et al. Nucleosome Patterns in Circulating Tumor DNA Reveal Transcriptional Regulation of Advanced Prostate Cancer Phenotypes, Cancer Discov 2023;13:632-653.
